# A New Black Carbon Sensor for Dense Air Quality Monitoring Networks

**DOI:** 10.3390/s18030738

**Published:** 2018-03-01

**Authors:** Julien J. Caubel, Troy E. Cados, Thomas W. Kirchstetter

**Affiliations:** 1 Department of Mechanical Engineering, University of California, Berkeley, CA 94720, USA; 2 Energy Technologies Area, Lawrence Berkeley National Laboratory, Berkeley, CA 94720, USA; tcados@lbl.gov (T.E.C.); twkirchstetter@lbl.gov (T.W.K.); 3 Department of Civil and Environmental Engineering, University of California, Berkeley, CA 94720, USA

**Keywords:** air quality monitoring, black carbon, wireless sensor network

## Abstract

Low-cost air pollution sensors are emerging and increasingly being deployed in densely distributed wireless networks that provide more spatial resolution than is typical in traditional monitoring of ambient air quality. However, a low-cost option to measure black carbon (BC)—a major component of particulate matter pollution associated with adverse human health risks—is missing. This paper presents a new BC sensor designed to fill this gap, the Aerosol Black Carbon Detector (ABCD), which incorporates a compact weatherproof enclosure, solar-powered rechargeable battery, and cellular communication to enable long-term, remote operation. This paper also demonstrates a data processing methodology that reduces the ABCD’s sensitivity to ambient temperature fluctuations, and therefore improves measurement performance in unconditioned operating environments (e.g., outdoors). A fleet of over 100 ABCDs was operated outdoors in collocation with a commercial BC instrument (Magee Scientific, Model AE33) housed inside a regulatory air quality monitoring station. The measurement performance of the 105 ABCDs is comparable to the AE33. The fleet-average precision and accuracy, expressed in terms of mean absolute percentage error, are 9.2 ± 0.8% (relative to the fleet average data) and 24.6 ± 0.9% (relative to the AE33 data), respectively (fleet-average ± 90% confidence interval).

## 1. Introduction

Air quality monitoring networks operated by regulatory agencies traditionally rely on a small number of measurement sites centrally located within large geographical areas. For example, the European Union only requires one sampling site to monitor an area of 100,000 km^2^ [1]. The number of monitoring sites is primarily restricted by the cost of expensive regulatory-grade air pollution analyzers housed in dedicated, environmentally controlled structures [2,3]. Monitoring at central locations is highly valuable for establishing air pollution concentration trends [4], but pollutant concentrations measured at a single location in a neighborhood or urban area do not necessarily accurately describe the pollution exposures of individuals located throughout that area [5,6]. This is particularly true for primary pollutants, whose concentrations tend to vary widely with location [7,8].

Emerging low-cost sensors offer the opportunity to monitor air pollution with much greater spatial resolution [9,10]. Low-cost sensors that cost a few hundred dollars or less are available for many pollutant gases, such as electrochemical sensors for nitrogen dioxide or ozone [11,12,13,14]. Low-cost sensors that measure particulate matter (PM) are also available, where mass concentration is typically based on the amount of light scattered by the airborne particles [15,16,17]. Combinations of these low-cost sensors are increasingly deployed in densely distributed sensor networks to provide greater spatial resolution than traditional regulatory monitoring networks [18,19,20,21,22]. One notable gap, however, is the absence of a black carbon (BC) sensor in these networks.

BC, a primary air pollutant, is the main light-absorbing component of PM generated by fossil fuel combustion (notably diesel engines) and biomass burning (such as woodstoves) [23]. Exposure to particulate matter from these sources is associated with increased risk of pulmonary and cardiovascular diseases, cancer, and premature death [24,25,26]. BC is also a potent short-lived climate pollutant [27,28]. BC analyzers employing different measurement principles exist [29,30]. However, most cost on the order of $10,000 to $20,000 (USD) and are thus too expensive to be deployed in large numbers.

This paper presents a new BC sensor—the Aerosol Black Carbon Detector (ABCD)—designed for dense deployment in air quality monitoring networks. In addition to providing an overview of the ABCD’s design architecture, this paper presents a detailed evaluation of the sensor’s native sensitivity to ambient temperature fluctuations, and demonstrates a novel data processing methodology to correct this temperature dependence. This data processing method greatly reduces inaccurate or erroneous BC measurements that plague other BC analyzers [31,32]. To validate the performance of this new sensor, we constructed over 100 ABCDs and operated this sensor fleet outdoors in collocation with a commercial BC instrument housed inside a regulatory air quality monitoring station in Oakland, California.

## 2. Materials and Methods

### 2.1. Aerosol Black Carbon Detector (ABCD)

**Figure 1:**
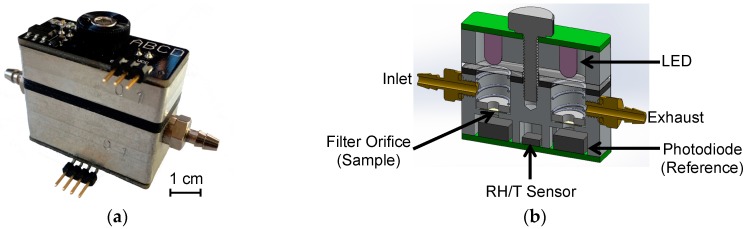
Aerosol Black Carbon Detector (ABCD): (**a**) Optical cell; (**b**) Section view of optical cell.

The ABCD belongs to a class of instruments known as aerosol absorption photometers, which include the particle soot absorption photometer, the aethalometer, and the multi-angle absorption photometer [33,34,35]. These instruments measure the light absorption of ambient PM collected on a fibrous filter. The ABCD converts measured light absorption to BC mass concentration in the sampled air flow. The central component of the ABCD is the optical cell, shown in Figure 1.

**Figure 2:**
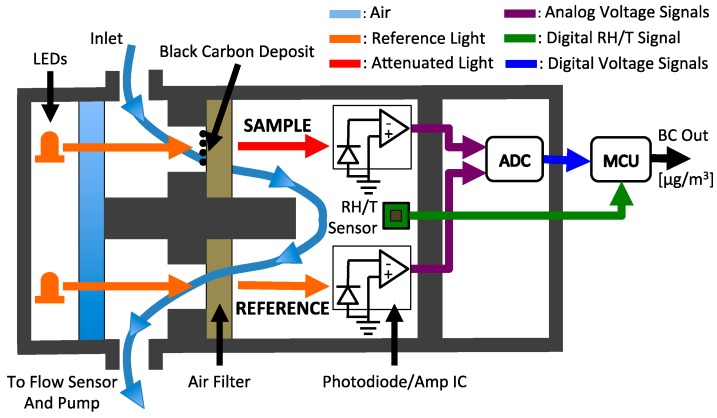
Functional diagram of the ABCD optical cell.

Air is drawn into the cell with a rotary vane pump, and through two Teflon-coated glass-fiber filters (Pallflex^®^ Emfab™) that lie between light emitting diodes (LEDs) and photodiodes, as illustrated in Figure 2. The LEDs operate at a central wavelength of 880 nm, where BC is the predominant PM species to absorb light [36,37,38,39]. The photodiodes generate electrical voltages that are linearly proportional to the intensity of light transmitted through each filter. The analog voltage measurements from the photodiodes are digitized using a 24-bit analog-to-digital converter (ADC) and processed by a microcontroller unit (MCU). A relative humidity and temperature (RH/T) sensor is mounted directly in the sample flow path between the sample and reference photodiodes. A differential pressure sensor downstream of the optical cell measures the volumetric flow rate of the sampled air (see Appendix D). The MCU generates a pulse-width modulated signal to control the electrical power delivered to the rotary vane pump and maintain a desired flow rate between 100 and 250 cc min^−1^.

As the ABCD samples polluted air, the intensity of light reaching the photodiode below the first filter in series (i.e., the sample filter) is attenuated by the accumulation of BC. Emfab™ filters have a particle removal efficiency of 99.9% [40], so the air reaching the second filter (i.e., the reference filter) does not contain any light-absorbing BC. Every two seconds, the ABCD’s MCU computes optical attenuation as the natural log ratio of the reference to sample voltage outputs (Equation (A1)) and calculates BC mass concentrations according to Equation (A2). Upon reaching an optical attenuation of roughly 100 units (indicating that the sample filter is highly loaded with BC), the Emfab™ filters in the optical cell are replaced to avoid potential optical saturation [41,42,43]. To further illustrate the ABCD’s principle of operation, Appendix C provides a representative set of ABCD output data collected during the loading of a single sample filter in the field.

Air is drawn through both the reference and sample filters with the intention of minimizing the influence of environmental conditions on measured BC concentrations, such as changes in the relative humidity of the sampled air and temperature of the electrical components. To further reduce the BC sensor’s environmental sensitivity, a real-time temperature compensation method was developed, as described in detail below.

**Figure 3:**
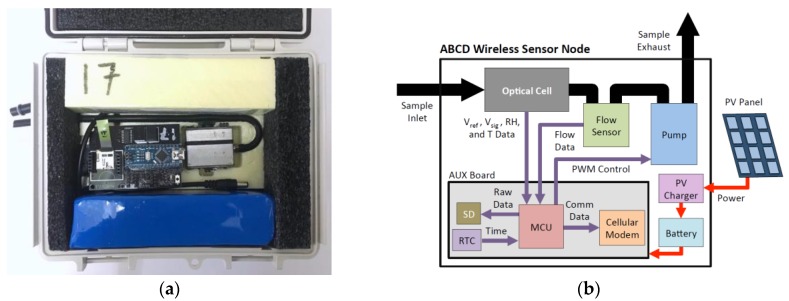
Complete ABCD outdoor deployment package: (**a**) Interior view; (**b**) Functional diagram.

The optical cell is enclosed with all the components required for the ABCD to serve as a self-contained, wireless sensor node, as shown in Figure 3. This outdoor deployment package includes rotary vane vacuum pump, air flow sensor, rechargeable battery (12-volt, 10-amp-hour), and an integrated electronics board, known as the AUX board. The AUX board is a printed circuit board that integrates the MCU, real-time clock, SD memory card slot, 2G cellular modem, power management electronics, and hardware for all required input and output connections. The enclosure is a weatherproof, insulated box onto which a photovoltaic panel (18-volt, 8-watt) is mounted. A charge controller regulates the power generated to recharge the battery. Additional details of these components are provided in Appendix E. The ABCD’s MCU processes and stores data to the SD memory card every 2 s, and for this study, the ABCD was programmed to wirelessly transmit 60 one-minute average values every hour to an online database using the 2G cellular modem. Appendix F provides details on the data collection and analysis methods.

The complete ABCD has dimensions of 18 cm × 23 cm × 10 cm, weighs 1.5 kg, and consumes 0.6 watt of electrical power at a sample flow rate of 110 cc min^−1^. Under this operating condition, the onboard battery can power the ABCD for ~8 days. The photovoltaic panel extends this operational period indefinitely if weather conditions and instrument placement allow sufficient insolation. Internal insulation is intended to prevent condensation when sampling cold, moist air by keeping the optical cell above the dew point temperature. The optical cell is positioned in the enclosure for easy removal and is readily opened using a single thumbscrew. These features allow for easy replacement of the sample filter after it becomes excessively loaded with BC.

The custom optical cell is designed with a minimal number of parts to simplify the fabrication process and reduce manufacturing costs. The MCU, flow sensor, charge controller, battery, and pump are commercially available components. This design approach enables the construction of a complete ABCD sensor at a material cost of roughly $400 for a production batch of around 150 units. The pump ($125) and custom optical cell ($100) account for about half of this cost (see Appendix E for a comprehensive list of components).

### 2.2 Field Validation

**Figure A1:**
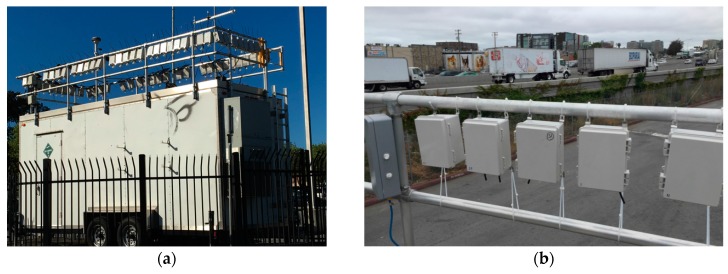
(**a**) Over 60 ABCDs hung from the roof railing of the Bay Area Air Quality Management District roadside monitoring station in Oakland, California; (**b**) Close up view of ABCDs deployed at the monitoring station, with California Highway 880 in the background.

ABCDs were deployed at an air quality monitoring station operated by the Bay Area Air Quality Management District. The station is adjacent to a major highway in Oakland, California, and houses an aethalometer (Model AE33, Magee Scientific, Berkeley, CA, USA) to measure ambient BC mass concentrations. While operating inside the station, the AE33 samples outside air using a probe that extends through the station’s roof. ABCDs were hung from railings on the station’s roof, as shown in Figure A1, and operated for about 1-week periods. BC concentrations measured with ABCD units were compared to those measured with the AE33.

## 3. Results

### 3.1. Measurement Bias from Environmental Fluctuations

**Figure 4:**
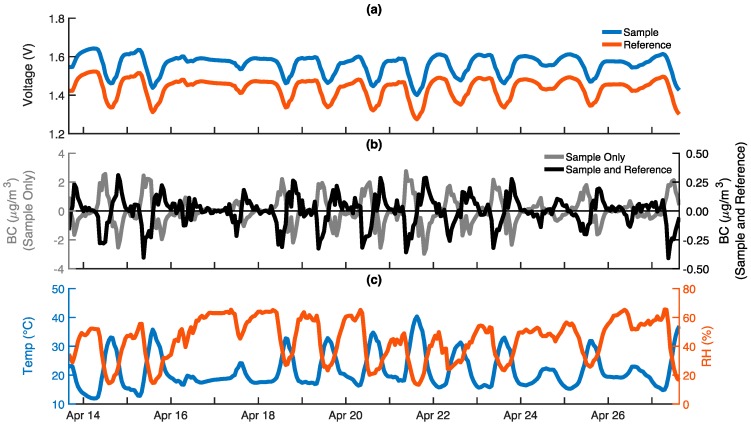
The response of an ABCD operating outdoors with a HEPA filter on its air inlet for two weeks: (**a**) Sample (blue) and reference (red) voltage outputs from optical cell; (**b**) Black carbon (BC) concentrations calculated using only sample voltage (gray), and using both the sample and reference voltages (black); (**c**) Optical cell temperature (blue) and relative humidity (RH) (red). All data is provided on a 60-m time base.

The response of an ABCD operating outdoors with a High Efficiency Particulate Air (HEPA) filter on its inlet for a two-week period is shown in Figure 4. The output voltages (Figure 4a) are clearly dependent on ambient conditions, oscillating in sync with the diurnal trends in temperature and relative humidity (Figure 4c). These output voltage oscillations are likely the result of the optical electronics’ temperature sensitivity. The LEDs are rated to dim 0.3% for every 1 °C temperature rise [44], which is approximately what is observed (0.1 V reduction relative to a 1.5 V baseline with a 20 °C temperature increase), suggesting that the temperature sensitivity of the LEDs plays a major role. Photodiode sensitivity (the voltage output per watt of incident light intensity) decreases by 0.01% for every 1 °C temperature rise [45], and likely also contributes to the diurnal voltage oscillations. It should be noted that the ABCD measures the temperature of the air flowing through the optical cell, but it is assumed that the electronics nearby are at a similar temperature. Expected variations in the optical thickness of the fibrous filters due to sorption and desorption of water vapor from the sampled air are opposite to the observed voltage oscillations, suggesting that RH sensitivity is smaller than the temperature dependence of the optical electronics.

Although the sample and reference output voltage oscillations track one another closely, the rates of voltage change over time are not identical. Consequently, reported BC concentrations are not zero, as would be expected for a sensor sampling particle-free air. Rather, BC concentrations exhibit a diurnal trend typically in the ± 0.3 μg m^−3^ range (Figure 4b, black), with a mean absolute error (MAE) on the order of 0.1 μg m^−3^ and a two-week average BC concentration of −0.003 μg m^−3^. BC concentrations computed using only the output from the photodiode monitoring the sample filter are much larger, in the ± 2 μg m^−3^ range (Figure 4b, gray), which illustrates that computing BC concentrations using both the reference and sample signals significantly reduces, but does not completely eliminate, the sensor’s sensitivity to environmental conditions. If ambient BC concentrations are much larger than ±0.3 μg m^−3^, then further compensation may not be necessary. However, in many locations, ambient BC concentrations are comparable to 0.3 μg m^−3^ and, thus, temperature compensation is employed to further reduce the environmental sensitivity.

### 3.2. Temperature Compensation

**Figure 5:**
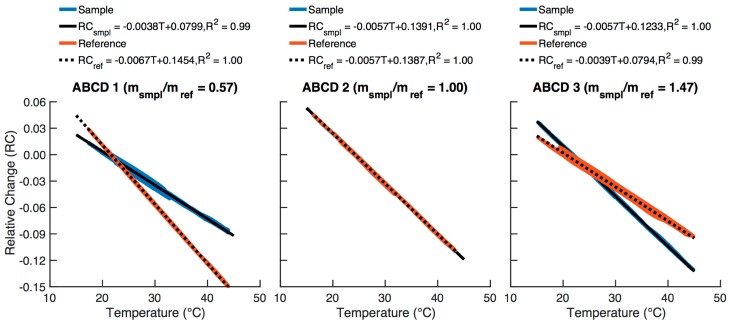
Relative change (RC) in the sample (blue) and reference (red) voltage outputs of three ABCD optical cells as a function of sample flow temperature, along with corresponding linear regression equations and coefficients of determination. The ratio of the sample to reference temperature sensitivities (m_smpl_/m_ref_) of each optical cell is noted above each plot.

The temperature response of each ABCD optical cell was determined by operating each instrument outdoors with a HEPA filter on the inlet for at least 24 h. In all cases, sample and reference photodiode voltage outputs display a highly linear dependence on the recorded cell temperature. In order to quantify this temperature dependence, the relative change (*RC*) in each photodiode’s output voltage is calculated as:

(1) $$RC\left(t\right)=\frac{V\left(t\right)-V\left(0\right)}{V\left(0\right)},$$

where *V*(*t*) is the photodiode voltage (V) at time *t*, and *V*(0) is the first voltage logged during the particle-free sampling event. In Figure 5, *RC* is plotted as a function of sensor temperature for three ABCD optical cells, and linear regression factors (slope, intercept, and R^2^) are shown. The temperature sensitivities of an optical cell’s sample and reference channels (i.e., the slopes of the linear regressions, m_smpl_ and m_ref_) are often not equal. Therefore, the ratio of these slopes (m_smpl_/m_ref_, hereafter referred to as “slope ratio”) is often either greater than or less than unity. For example, ABCD 1 has a slope ratio of 0.57, indicating that the sample voltage output is less temperature sensitive than the reference. Consequently, as the temperature fluctuates over time, the sample and reference voltage outputs do not change at an equal rate. The result is non-zero BC measurements, as the effect of changing temperature on the sample voltage output is not exactly compensated by the effect of changing temperature on the reference voltage output. For example, the optical cell referenced in Figure 4 has a slope ratio of 0.89, and the reference voltage output significantly reduces, but does not completely eliminate, the environmental influence on reported BC concentrations.

In Equation (2), the linear regression equations for each photodiode output are set equal to Equation (1), except that the voltage change is now evaluated relative to the temperature-compensated voltage:

(2) $$RC\left(t\right)=\frac{V\left(t\right)-V_{comp}\left(t\right)}{V_{comp}\left(t\right)}=mT\left(t\right)+b,$$

where *V_comp_*(*t*) is the temperature-compensated voltage output (*V*), *T*(*t*) is the sample flow temperature (°C), and *m* (°C^−1^) and *b* are the slope and intercept of the linear regression, respectively. Rearranging Equation (2) yields an equation that allows the photodiode voltage, *V*(*t*), to be compensated using real-time temperature measurements:

(3) $$V_{comp}\left(t\right)=\frac{V\left(t\right)}{mT\left(t\right)+b+1}.$$

BC concentrations calculated using the temperature-compensated sample and reference voltage outputs from Equation (3) are generally significantly less sensitive to temperature fluctuations.

We observed considerable variability in the temperature sensitivity of optical cells (e.g., as illustrated in Figure 5), likely because of variations in the LEDs, photodiodes, and related circuitry. Consequently, we evaluated the temperature sensitivity and determined the linear regression coefficients in Equation (3) uniquely for each individual ABCD optical cell. The slope and intercept for both photodiode outputs are stored on SD cards assigned to each optical cell. The SD card is inserted into the ABCD’s AUX board, and the respective linear regression coefficients are uploaded to the MCU to compensate BC measurements in real time as a function of measured temperature.

**Figure 6:**
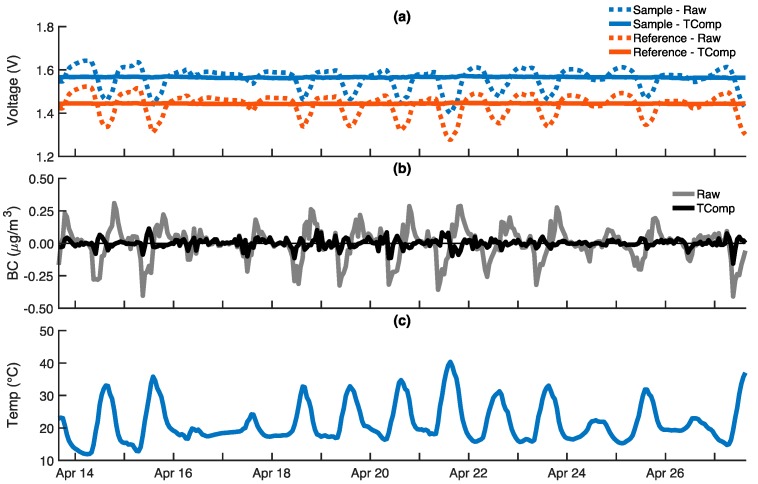
The response of an ABCD operating outdoors with a HEPA filter on its air inlet for two weeks. Temperature-compensated responses (solid) are shown in addition to the uncompensated responses (dashed) previously provided in Figure 4: (**a**) Sample (blue) and reference (red) voltage outputs from optical cell; (**b**) Uncompensated (gray) and temperature-compensated (black) black carbon (BC) concentrations; (**c**) Optical cell temperature. All data is provided on a 60-min time base.

In Figure 6, temperature-compensated (TComp) responses are shown in addition to the uncompensated (Raw) responses for the ABCD shown in Figure 4. Throughout the trial, temperature-compensated voltage outputs steadily maintain their initial values (Figure 6a) and temperature-compensated BC concentrations (Figure 6b) exhibit a diurnal trend typically in the ±0.1 μg m^−3^ range (compared to ±0.3 μg m^−3^ when uncompensated) with an MAE of 0.02 μg m^−3^ (compared to 0.1 μg m^−3^ when uncompensated).

**Figure 7:**
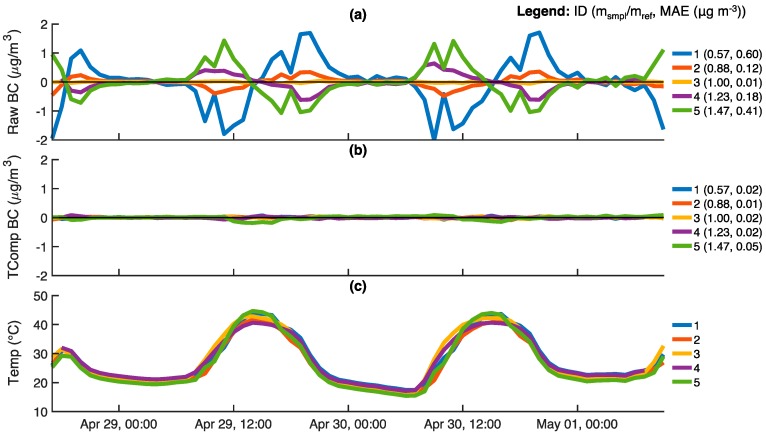
The response of five ABCD units operating outdoors with HEPA filters on the air inlets: (**a**) Uncompensated black carbon (BC) concentrations; (**b**) Temperature-compensated BC concentrations; (**c**) Optical cell temperatures. All data is provided on a 60-min time base. For each ABCD, the slope ratio of the optical cell and mean absolute error (MAE) of BC concentration measurements are shown in the legends. The MAE of BC measurements is evaluated relative to the desired zero response (0 μg m^−3^).

To further illustrate the implementation of the temperature compensation method, both uncompensated and temperature-compensated BC concentrations are shown in Figure 7 for five ABCDs with HEPA-filtered inlets. The slope ratios for these cells range from 0.57 to 1.47 and uncompensated BC concentrations ranged between ±2 μg m^−3^. As shown in Figure 7a, uncompensated diurnal BC oscillations and corresponding MAE values are largest for ABCD optical cells whose slope ratios are farthest from unity (cells 1 and 5) and smallest for the cell with a slope ratio of 1.00 (cell 3). Furthermore, as a consequence of the temperature dependence illustrated in Figure 5, BC oscillations for optical cells with slope ratios less than unity (cells 1 and 2) are opposite those for optical cells with slope ratio greater than unity (cells 4 and 5).

As shown in Figure 7b, all temperature-compensated BC concentrations are very close to the true zero, with MAE values on the order of 0.02 μg m^−3^ irrespective of the optical cell’s slope ratio. BC concentrations and MAE for cell 3 whose slope ratio is ~1 are essentially unaltered by the procedure, as expected.

**Figure 8:**
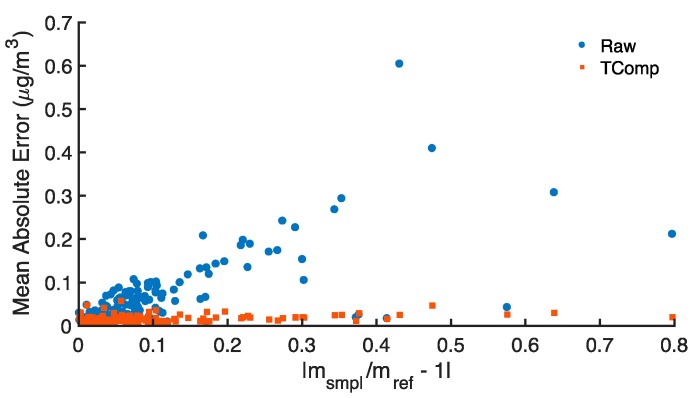
Mean absolute error (MAE) of uncompensated (Raw) and temperature-compensated (TComp) black carbon (BC) concentrations reported by 150 ABCD optical cells with HEPA-filtered inlets, as a function of the slope ratio’s absolute deviation from unity (|m_smpl_/m_ref_ − 1|). The MAE of 60-min time base BC concentrations is evaluated relative to the desired zero response (0 μg m^−3^).

Figure 8 summarizes the zero response of all 150 ABCD optical cells manufactured in this study. MAEs of uncompensated and temperature-compensated BC concentrations are plotted against the absolute deviation of each cell’s slope ratio from unity (|m_smpl_/m_ref_ − 1|). The figure illustrates that uncompensated MAE generally increases proportionally with increasing absolute slope ratio deviation from unity. In contrast, temperature compensation works to significantly improve performance: all ABCD optical cells report BC concentrations near zero such that, across the fleet of 150 cells, the temperature-compensated MAE averages 0.016 ± 0.001 μg m^−3^ (mean ± 90% confidence interval).

### 3.3. Field Validation

**Figure 9:**
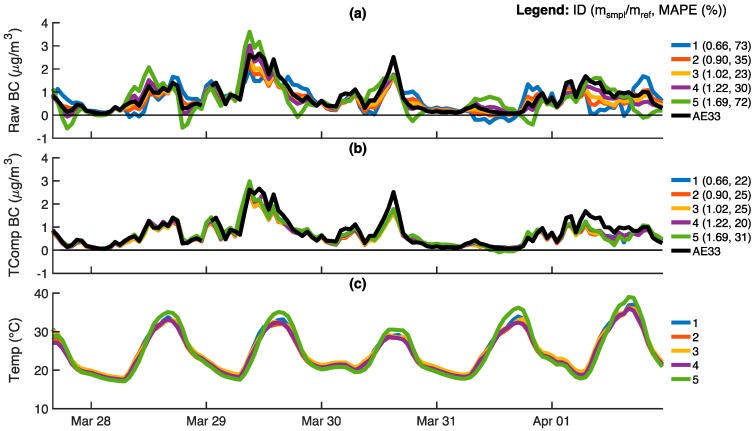
Five ABCD units operating outdoors atop a Bay Area Air Quality Management District near-roadway monitoring station: (**a**) Uncompensated black carbon (BC) concentrations; (**b**) Temperature-compensated BC concentrations; (**c**) Optical cell temperatures. Also shown are BC concentrations reported by a Magee Scientific AE33 housed inside the monitoring station (black). All data is provided on a 60-min time base. For each ABCD, the legend shows the slope ratio of the optical cell and mean absolute percent error (MAPE) of the BC measurements, the latter of which is based on deviations from BC concentrations reported by the AE33 reference instrument. The five ABCDs shown in this figure are different than those presented in Figure 7.

Following acquisition of temperature compensation parameters for all optical cells, ABCDs were operated outdoors atop the Bay Area Air Quality Management District’s near-roadway monitoring station (see Figure A1). Figure 9 shows time series of uncompensated and temperature-compensated BC concentrations for five ABCDs with optical cell slope ratios ranging from 0.66 to 1.69. BC concentrations reported by the aethalometer (Magee Scientific, Model AE33) housed inside the monitoring station are also shown for comparison.

Uncompensated BC concentrations deviate more notably from the AE33 reference for ABCD optical cells with slope ratios that are significantly offset from unity (Figure 9a). Uncompensated BC concentrations include erroneous negative values over substantial portions of the sampling period. For ABCD optical cells with slope ratios of 0.66 and 1.69, the corresponding mean absolute percent error (MAPE) in BC concentration relative to the AE33 (~70%) is about three times larger than the MAPE for the ABCD optical cell with a slope ratio of 1.02 (23%). Furthermore, optical cells with slope ratios less than unity tend to overestimate BC concentrations when the temperature increases (and vice-versa). Temperature compensation reduces measurement bias (Figure 9b). The five ABCDs have temperature-compensated MAPEs ranging from 22% to 31%, and negative BC measurements are nearly eliminated.

**Figure 10:**
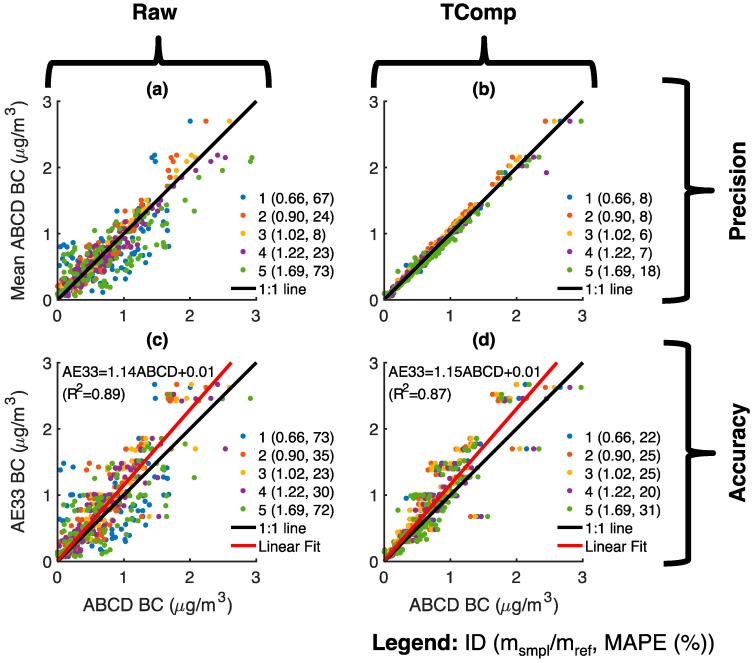
Five ABCD units operating atop a Bay Area Air Quality Management District near-roadway monitoring station: (**a**) Precision of uncompensated (Raw) black carbon (BC) concentrations; (**b**) Precision of temperature-compensated (TComp) BC concentrations; (**c**) Accuracy of uncompensated BC concentrations; (**d**) Accuracy of temperature-compensated BC concentrations. All data is provided on a 60-min time base. Precision is evaluated relative to the mean of BC measurements from all 5 ABCD units, while accuracy is evaluated relative to the AE33 reference instrument. Accuracy plots (**c**,**d**) also provide the least-square linear regression of the aggregate ABCD data to the AE33.

Figure 10 shows the precision and accuracy of these ABCDs during the field evaluation period. The precision of each ABCD is evaluated relative to mean BC concentrations from the fleet of five ABCDs, while accuracy is evaluated relative to the AE33. Sensor precision (compare Figure 10a,b) and accuracy (compare Figure 10c,d) are much improved through the temperature compensation method. Temperature-compensated data are less scattered and have lower MAPEs. For example, uncompensated BC concentrations from optical cells with slope ratios farthest from unity have MAPEs of ~70% relative to both the ABCD fleet average and the AE33 reference. In contrast, most temperature-compensated ABCDs have a precision error of ~8% and accuracy error of ~25%. However, temperature-compensated hourly data from ABCD 5, whose optical cell slope ratio is 1.69, still contain a few negative BC measurements that significantly increase both the precision and accuracy error. This suggests that the method presented does not fully compensate the temperature dependence of optical cells with slope ratios that deviate excessively from unity. On the other hand, the precision and accuracy of ABCD 3, whose optical cell slope ratio is 1.02, are essentially unaltered by temperature compensation, as expected.

**Figure 11:**
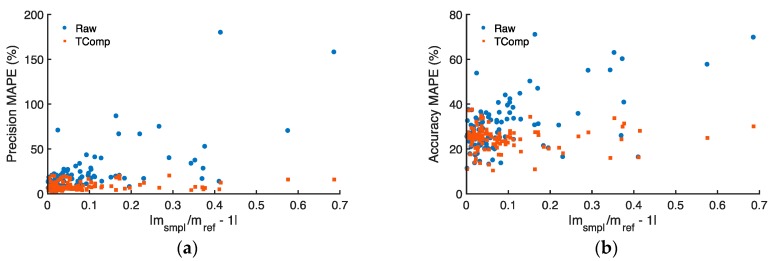
Mean absolute percentage error (MAPE) of uncompensated (Raw) and temperature-compensated (TComp) black carbon (BC) concentrations reported during field operation of 105 ABCD optical cells, as a function of the slope ratio’s absolute deviation from unity (|m_smpl_/m_ref_ − 1|): (**a**) Precision MAPE evaluated relative to the mean ABCD response; (**b**) Accuracy MAPE evaluated relative to the AE33 reference instrument.

The field performance of 105 ABCD optical cells is plotted in Figure 11 as a function of the slope ratios’ absolute deviation from unity (|m_smpl_/m_ref_ − 1|). The precision and accuracy of uncompensated BC concentrations diminish as the slope ratio increasingly deviates from unity, but temperature compensation generally improves measurement performance throughout. Similarly to the ABCDs featured in Figure 10, the temperature-compensated fleet-average precision and accuracy MAPEs of the 105 ABCD optical cells are 9.2 ± 0.8% and 24.6 ± 0.9%, respectively (mean ± 90% confidence interval).

Uncompensated and temperature-compensated ABCD BC concentrations are ~15% lower than those reported by the AE33 (see linear regressions in Figure 10c,d), and even after temperature compensation, the MAPEs of most ABCDs are above 20% relative to the AE33 (Figure 11b). This bias may be related to the value chosen to convert ABCD optical absorption to BC mass concentration (i.e., the mass attenuation coefficient in Equation (A2)) or the so-called “loading artifact”. The loading artifact causes underestimation of BC concentrations with increased loading of the sample filter [41,42,43]. Whereas the AE33 periodically changes its filter and incorporates a software algorithm to correct for the loading artifact [46], the ABCDs in this study are operated using only a single set of filters for each trial, and the data presented here have not been adjusted for a loading artifact.

## 4. Discussion and Conclusions

**Figure A5:**
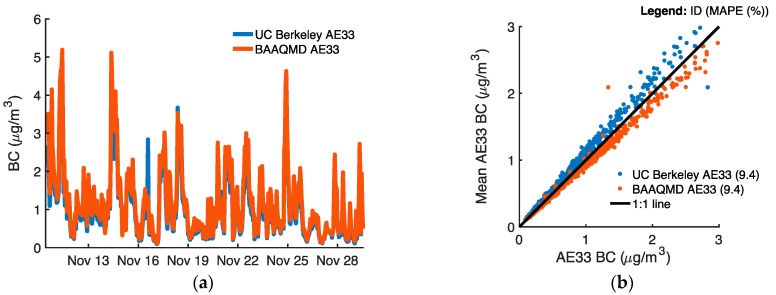
Two Magee Scientific AE33 instruments operating inside the Bay Area Air Quality Management District roadside monitoring station: (**a**) Time series of black carbon (BC) mass concentration measurements; (**b**) Precision of BC mass concentration measurements. All data is provided on a 60-min time base. Precision error is evaluated relative to the mean of BC measurements from both AE33 instruments, and is provided in the legend.

The ABCD’s compact weatherproof enclosure, solar-powered rechargeable battery, and cellular communication enable its remote operation over extended periods. The temperature compensation method presented in this study significantly diminishes the ABCD’s sensitivity to varying ambient temperature (with internal sensor temperatures spanning 15 °C to 45 °C), thereby increasing the precision and accuracy of measured BC mass concentrations in unconditioned indoor and outdoor operating environments. ABCDs deployed outdoors at a Bay Area Air Quality Management District monitoring station demonstrate a precision error of ~9% and an accuracy error of ~25% when evaluated relative to the commercial instrument (Magee Scientific, Model AE33). To provide a benchmark against which to compare this measurement performance, two AE33 instruments were operated side-by-side in the monitoring station for 20 days (see Figure A5). During this period, the precision error of the AE33 instruments was ~9% (the MAPE of each AE33 relative to the mean of both instrument measurements). Therefore, the ABCD’s measurement precision, while operating outdoors under fluctuating ambient conditions, generally matches that of the commercial instrument operating inside the monitoring station. The temperature compensation method could be further validated under a wider range of environmental conditions.

Ongoing research includes comparison of the ABCD and AE33 instruments at additional sampling locations, further investigation of the loading artifact, and a citywide deployment of the ABCDs in an air quality monitoring network to provide insights into BC emission sources and spatiotemporal patterns in BC mass concentrations.

## Figures and Tables

**Figure A2:**
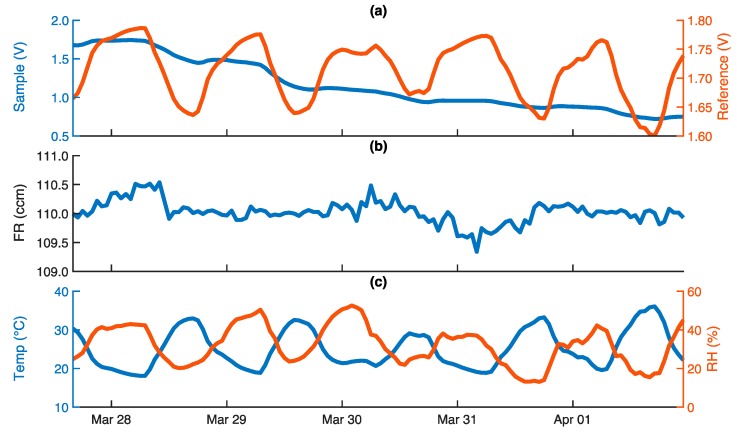
Uncompensated (raw) data measurements collected by an ABCD operating outdoors: (**a**) Sample and reference voltage signals; (**b**) Sample flow rate; (**c**) Temperature and relative humidity (RH). All data is provided on a 60-min time base.

**Figure A3:**
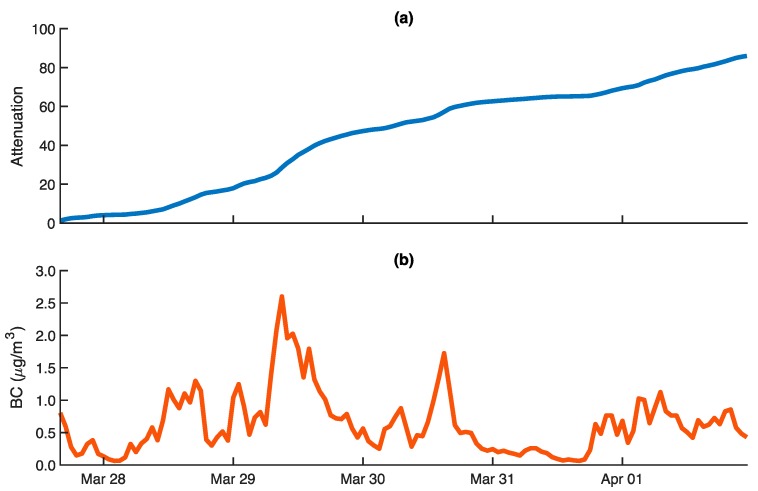
Temperature-compensated data calculated by an ABCD operating outdoors: (**a**) Optical attenuation; (**b**) Black carbon (BC) mass concentration. All data is provided on a 60-min time base.

**Figure A4:**
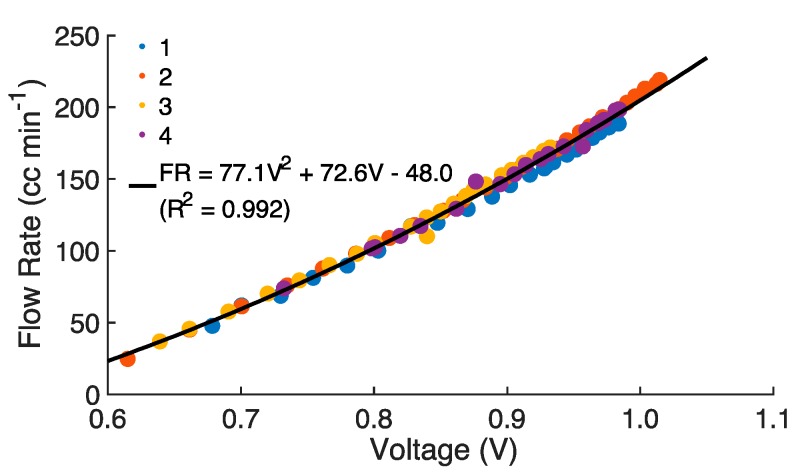
Flow sensor calibration data. Volumetric flow rate of air sampled through ABCD plotted as function of voltage output from four different Omron D6F differential pressure sensor units. A quadratic regression equation is derived for the aggregate data collected from all four sensors, and is shown with the corresponding coefficient of determination (R^2^).

## Appendix A

The Aerosol Black Carbon Detector (ABCD) calculates optical attenuation (*ATN*) as:

(A1) $$ATN\left(t\right)=100\cdot \ln\left(\frac{V_{ref}\left(t\right)}{V_{smpl}\left(t\right)}\cdot \frac{V_{smpl}\left(0\right)}{V_{ref}\left(0\right)}\right),$$

where *V_smpl_*(*t*) and *V_ref_*(*t*) (V) are the optical cell’s sample and reference voltage outputs at each measurement time step ‘*t*’, respectively. Note that *V_smpl_*(0) and *V_ref_*(0) denote the voltage outputs recorded prior to the first measurement time step (when the instrument is first turned on with clean filters). Black carbon (*BC*) mass concentrations (μg m^−3^) at each measurement time interval are calculated based on optical attenuation and flow rate as:

(A2) $$BC\left(t_{i}\right)=\frac{A}{Q\left(t_{i}\right)\cdot MAC\cdot \left(t_{i}-t_{i-1}\right)}\cdot \ln\left(\frac{ATN\left(t_{i}\right)}{ATN\left(t_{i-1}\right)}\right)=\frac{A}{Q\left(t_{i}\right)\cdot MAC\cdot \left(t_{i}-t_{i-1}\right)}\cdot \ln\left(\frac{V_{ref}\left(t_{i}\right)}{V_{smpl}\left(t_{i}\right)}\cdot \frac{V_{smpl}\left(t_{i-1}\right)}{V_{ref}\left(t_{i-1}\right)}\right).$$

where *t_i-1_* and *t_i_* are the time stamps of two consecutive measurements, and *A* (m^2^) is the surface area of each filter orifice. In the ABCD optical cell, both circular orifices have a diameter of 0.003175 m (0.125 in), and corresponding area of 7.87 × 10^−6^ m^2^. *Q* (m^3^ s^−1^) is the volumetric flow rate of air through the instrument, as measured by the differential pressure sensor downstream of the optical cell (see Appendix D). The ABCD’s flow rate is set between 1.67 × 10^−6^ and 4.17 × 10^−6^ m^3^ s^−1^ (100 and 250 cc min^−1^), and held constant. *MAC* (m^2^ g^−1^) is the mass attenuation coefficient of BC, which is set at 12.5 m^2^ g^−1^. The ABCD uses Teflon-coated glass filters (Pallflex^®^ Emfab™) and LEDs centered at a wavelength of 880 nm. Other BC instruments using similar fibrous filter materials at this incident wavelength use the same MAC value [47]. Throughout this study, the ABCD operated at a sampling frequency of 0.5 Hz, so Δ*t* = *t_i_ − t_i-1_* = 2 s.

## Appendix B

During field validation, ABCDs were deployed on the roof of the Bay Area Air Quality Management District roadside monitoring station, located near Laney College and Highway 880 in Oakland, California (as shown in Figure A1).

## Appendix C

The figures below provide a representative data set collected by an ABCD operating outdoors at the Bay Area Air Quality Management District monitoring station (see Appendix B). Figure A2 shows all uncompensated (raw) measurements collected by the ABCD: sample and reference voltage (Figure A2a), sample flow rate (Figure A2b), and temperature and relative humidity of the sampled flow (Figure A2c). Uncompensated voltages display diurnal oscillations. The sample voltage attenuates appreciably over the course of the trial as optically absorbing BC accumulates on the sample filter. The flow rate is set to 110 cc min^−1^ and held constant throughout.

Figure A3 provides the temperature-compensated optical attenuation and BC mass concentration data calculated onboard the ABCD (by the MCU) using the measurements shown in Figure A2. The optical attenuation reaches nearly 100 units over the 5-day sampling period. All data is provided on an hourly time base and filtered according to the procedures outlined in Appendix F.

## Appendix D

The flow rate of air sampled through the Aerosol Black Carbon Detector (ABCD) is measured using an Omron D6F differential pressure sensor installed inline between the optical cell and vacuum pump (as illustrated in Figure 3). The sensor outputs an analog voltage that is dependent on the volumetric flow rate of air through the sensor. The MCU digitizes the analog voltage using an integrated, 10-bit analog-to-digital converter (ADC), so it can be used to calculate BC mass concentrations. To calibrate the differential pressure sensor, the analog voltage output is recorded while the ABCD’s sample flow rate is simultaneously measured (at the inlet, with clean filters loaded in the optical cell) with a bubble meter (Gilian Gilibrator). Flow calibration data was collected using four different flow sensor units, as shown in Figure A4, and a least-squares regression was used to generate the quadratic calibration equation:

(A3) $$FR=77.1{\left(V_{FR}\right)}^{2}+72.6\left(V_{FR}\right)-48.0.$$

where *FR* (cc min^−1^) is the sample flow rate at the inlet, and *V_FR_* (V) is the analog voltage output by the differential pressure sensor. Figure A4 shows that the calibration data is repeatable between flow sensor units, and that the empirical equation generated closely fits the aggregate data set, with a coefficient of determination (R^2^) of 0.992.

## Appendix E

Table A1 below lists the major components shown in the ABCD functional diagram (Figure 3), the corresponding material cost, and the component manufacturer and model where applicable. The prices listed represent the approximate cost of each component when ordering 150 units, and do not reflect the costs associated with the assembly, calibration, and validation of each ABCD. The total material cost of the components listed is $430.

**Table A1:** Major components of the Aerosol Black Carbon Detector.

Component	Manufacturer/Model	Approximate Material Cost (USD)
Optical Cell	N/A	100
AUX Board	N/A	80
Pump	Schwartzer/135 FZ	125
Flow Sensor	Omron/D6F	40
Battery	KXD/12 V, 10 AH	35
Photovoltaic Panel	Shine Solar/18 V, 8 W	20
Packaging	BUD Industries/NBF-32002	20
Miscellaneous ^1^	N/A	10

^1^ Miscellaneous components include electrical connectors, packaging insulation, and other minor items.

## Appendix F

The ABCD generates BC data that is stored directly on the onboard memory card (SD card) every 2 s. After zero-response trials, 2-s data is collected from the SD cards and used for temperature compensation calculations, without any data correction or filtering. During field validation, the ABCD transmits one-minute time base data to an online server every hour, where it is cataloged and stored. SD card data is also collected manually to supplement the wireless data set during periods when the sensor is operating but unable to communicate with the online server. The 2-s time base data from the SD card is averaged down to a one-minute time base (with time stamps synchronized to calendar minutes) and concatenated with the wireless data set downloaded from the online server.

The aggregated field validation data is filtered to remove erroneous measurements resulting from hardware errors or unsuitable operating conditions. Three data filters are implemented on the one-minute time base data:

1. **BC Outlier Filter:** Remove all data points where the absolute value of the BC measurement is greater than 100 μg m^−3^. BC concentrations on this order of magnitude are improbable while sampling ambient air, and instead usually result from hardware errors, such as disconnection or disturbance of the optical cell during field maintenance (changing of filters or batteries).
2. **High Attenuation Filter:** Remove data when measured optical attenuation is greater than 100 units to avoid possible optical saturation effects.
3. **Flow Rate Filter:** Remove all data generated when the ABCD is operating at a flow rate that deviates by more than 5 cc min^−1^ from the nominal set point (usually 110 cc min^−1^). It was found that some rotary vane pumps failed during deployment, so the flow rate through the ABCD could not be steadily maintained. Consequently, all data with non-nominal sample flow is discarded to account for pump failures and potentially inaccurate flow rate measurements.

Hourly average values are calculated by taking the simple mean of all filtered one-minute measurements collected within a calendar hour. Hourly average values calculated using less than 48 measurements (less than 80% of the 60 one-minute time base measurements that should ideally be collected every hour) are discarded. This filtering accounts for periods when the ABCD is operating intermittently, and may consequently provide inaccurate or erroneous data.

The ABCD’s performance is quantified using hourly average BC measurements. The Mean Absolute Error (MAE (μg m^−3^)) of hourly BC measurements collected when sampling with HEPA-filtered inlets is calculated as:

(A4) $$MAE=\;\frac{\sum_{t_{i}}^{t_{f}}\left|BC\left(t\right)-BC_{ref}\right|}{N}=\frac{\sum_{t_{i}}^{t_{f}}\left|BC\left(t\right)\right|}{N},$$

where *BC*(*t*) (μg m^−3^) represents hourly BC concentrations during the zero-response tests, from *t* = *t_i_* to *t* = *t_f_*, and N is the total number of hourly measurements collected. Since the ABCD is operated with a filtered inlet, the reference measurement (*BC_ref_* (μg m^−3^)) is 0 μg m^−3^ throughout, and the MAE expression reduces to the simple mean of the absolute BC data.

During field validation, the ABCDs are operated in collocation with a commercial BC instrument, the Magee Scientific AE33. The Mean Absolute Percent Error (MAPE (%)) of hourly BC measurements from the ABCD is evaluated relative to hourly reference measurements from the Magee Scientific AE33 (*BC_AE33_*(*t*) (μg m^−3^)):

(A5) $$MAPE=\frac{100}{N}\;\sum_{t_{i}}^{t_{f}}\left|\frac{BC\left(t\right)-BC_{AE33}\left(t\right)}{BC_{AE33}\left(t\right)}\right|,$$

where *BC*(*t*) (μg m^−3^) represents hourly BC concentrations during the field validation, from *t* = *t_i_* to *t* = *t_f_*, and *N* is the total number of hourly measurements collected.

## Appendix G

Over a period of 20 days, two Magee Scientific AE33 instruments were operated side-by-side inside the Bay Area Air Quality Management District monitoring station in Oakland, California. The instruments sampled ambient air at the same flow rate through two adjacent inlets extending out of the monitoring station roof. Figure A5a provides a time series of the black carbon (BC) mass concentration data collected, and Figure A5b is a scatter plot that illustrates precision. Over the 20-day period, both instruments exhibit a mean absolute percent error (MAPE) of roughly 9% relative to the mean of their measurements.
